# An annular eruption during pregnancy: Purpura annularis telangiectodes of Majocchi with atrophic features

**DOI:** 10.1016/j.jdcr.2026.07.032

**Published:** 2026-07-24

**Authors:** Shabnam Afzal, Deena Fayyad, Perya Bhagchandani, Louis J. Born, Mary Elizabeth Shenk, Emily R. Nadelmann

**Affiliations:** a Department of Dermatology, University of Maryland School of Medicine, Baltimore, Maryland; b Department of Dermatology, George Washington University School of Medicine and Health Sciences, Washington, District of Columbia

**Keywords:** capillaritis, hemosiderin, Majocchi disease, pigmented purpuric dermatosis, purpura annularis telangiectodes of Majocchi

## Introduction

Pigmented purpuric dermatoses (PPDs) are a group of benign vascular disorders that most often involve the lower extremities and present with petechiae or purpura with brown pigmentation related to hemosiderin deposition from iron breakdown.^1^^,^^2^ Histopathology typically demonstrates erythrocyte extravasation with superficial dermal hemosiderin and a mild superficial perivascular lymphocytic infiltrate.^1^^,^^2^ Purpura annularis telangiectodes of Majocchi is an uncommon variant that has been reported more frequently in young women and clinically presents with annular or geographic hyperpigmented patches and plaques on the legs.^3^ We report a case with onset during pregnancy and highlight the role of biopsy and clinicopathologic correlation to confirm diagnosis.

## Case report

A 29-year-old female presented for evaluation of asymptomatic hyperpigmented lesions on the bilateral lower extremities and feet. The eruption began during pregnancy 18 months prior as small erythematous and hyperpigmented patches that rapidly enlarged and darkened, then stabilized without resolution. She denied pruritus, pain, burning, or systemic symptoms. There was no personal or family history of similar eruptions. Treatment with over-the-counter emollients was ineffective. Physical examination revealed multiple well-demarcated hyperpigmented to violaceous, mildly atrophic annular plaques in a patchy distribution on the bilateral lower legs and feet (Fig 1, *A* and *B*).

**Fig 1:**
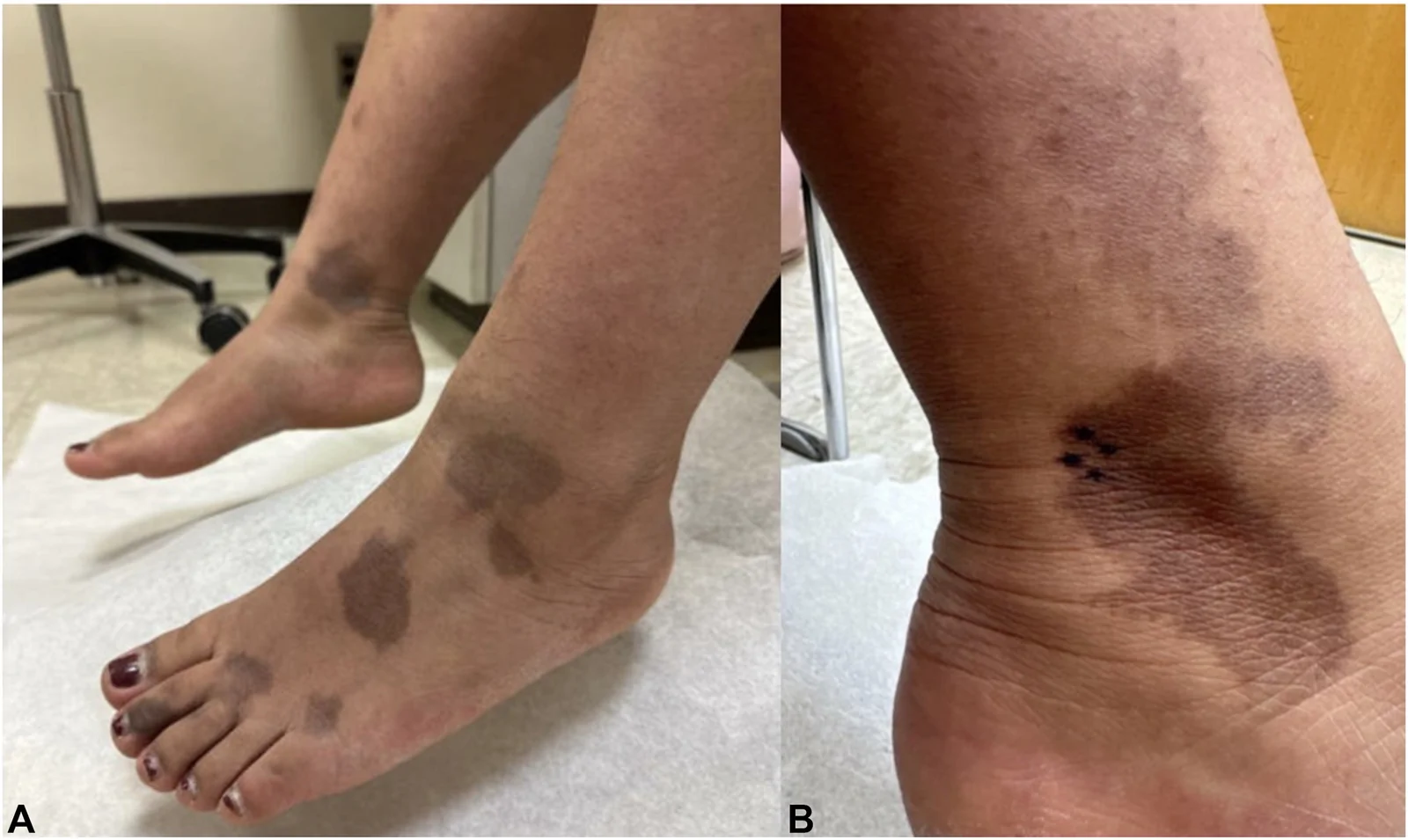
Pigmented purpuric dermatosis of the dorsal foot and lateral ankle. **A,** Brown patches involving the dorsal foot and ankle. **B,** Closer view of the lateral ankle.

Given concern for an inflammatory or fibrosing dermatosis, a punch biopsy of the left medial ankle was performed. Histopathologic examination demonstrated erythrocyte extravasation within the papillary dermis, hemosiderin deposition in the papillary dermis, and an associated superficial perivascular mononuclear infiltrate (Fig 2, *A* and *B*). A Perls stain highlighted superficial iron deposition, consistent with hemosiderin. In correlation with the clinical morphology, the histologic findings were most consistent with pigmented purpuric dermatosis, specifically purpura annularis telangiectodes of Majocchi.

**Fig 2:**
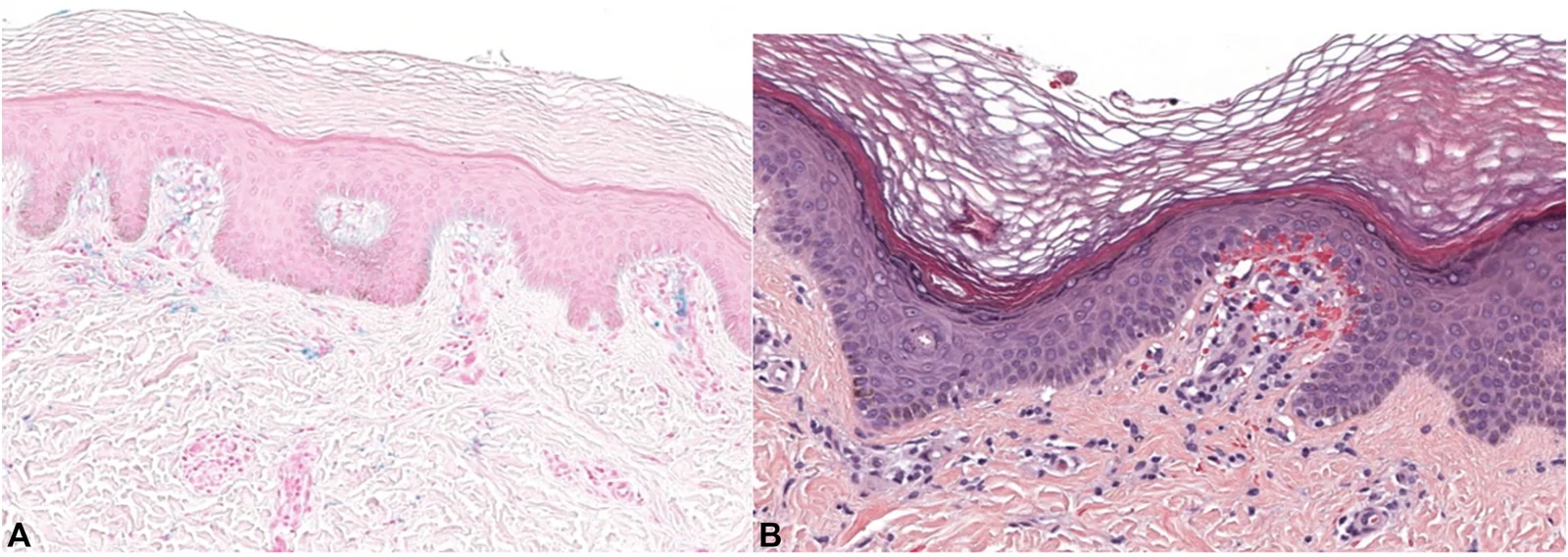
Pigmented purpuric dermatosis. **A,** Superficial perivascular lymphocytic infiltrate with erythrocyte extravasation and hemosiderin-laden macrophages within the papillary dermis (hematoxylin-eosin, original magnification ×10). **B,** Blue granular staining within dermal macrophages confirming hemosiderin deposition (iron stain, original magnification ×10).

The patient was initiated on triamcinolone 0.1% ointment applied twice daily to affected areas for up to 2 weeks with a 1-week interval off therapy. She was also started on oral rutoside 50 mg twice daily and vitamin C 500 mg twice daily.

## Discussion

PPDs are benign vascular disorders that typically affect the lower extremities and are characterized histologically by erythrocyte extravasation, hemosiderin deposition, and a mild superficial perivascular inflammatory infiltrate.^1^^,^^2^ Clinically, lesions may appear purpuric or erythematous and often develop brown pigmentation related to hemosiderin.^1^ In our patient, the eruption began during pregnancy as small erythematous patches that rapidly expanded and darkened, ultimately presenting as multiple hyperpigmented, mildly atrophic plaques on the bilateral lower extremities and feet. The absence of pruritus, pain, or burning is consistent with the typically asymptomatic course of PPD.

Accurate diagnosis required careful clinicopathologic correlation. The annular morphology and subtle atrophy raised concern for inflammatory or fibrosing dermatoses, including lichen planus and morphea. However, biopsy demonstrated papillary dermal erythrocyte extravasation with prominent superficial dermal hemosiderin and only a subtle superficial perivascular mononuclear infiltrate, without interface change or sclerosis. These findings favored PPD, specifically purpura annularis telangiectodes of Majocchi.

Majocchi-type PPD is an uncommon variant that has been described more often in young women and can present with annular or geographic hyperpigmented lesions on the legs, matching our patient’s morphology and distribution.^3^

The onset during pregnancy is notable. Although PPD is generally regarded as idiopathic, several subtypes have been reported in association with specific clinical contexts. Schamberg disease, the most common subtype, has been described in association with gravitational dependency, medication exposure, alcohol intake, and immune dysregulation, though many cases remain without a clear precipitating factor.^4^ In contrast, granulomatous PPD has been linked to hyperlipidemia and autoimmune comorbidities in a substantial proportion of reported cases, suggesting that systemic inflammatory or metabolic states may influence disease expression.^5^ Across subtypes, histopathology consistently demonstrates erythrocyte extravasation with a superficial perivascular inflammatory infiltrate, supporting a shared process of capillary leakage with secondary inflammation.^2^ Pregnancy may represent a physiologic state that lowers the threshold for capillary fragility and leakage. Pregnancy is associated with hormonal and hemodynamic changes that can alter endothelial function and increase lower extremity venous pressure, which may amplify microvascular permeability in predisposed patients.^6^ For our patient, the onset during pregnancy, rapid early evolution, and persistence postpartum support the possibility that pregnancy-related vascular changes served as a potential trigger.

Treatment of PPD can be frustrating for both patients and clinicians as lesions may persist for months to years.^2^ We initiated intermittent topical triamcinolone 0.1% ointment and oral rutoside with vitamin C, aiming to reduce any active inflammatory component while supporting capillary stability. Oral rutoside and ascorbic acid have been reported as a well-tolerated option with clinical improvement in progressive PPD (Schamberg type), though data specific to Majocchi variant are limited.^7^ This low-risk regimen was selected with counseling that gradual improvement, rather than complete clearance, is most realistic.

This case highlights Majocchi-type PPD with onset during pregnancy and emphasizes the role of biopsy in distinguishing PPD from other inflammatory or fibrosing mimickers. Recognition of this benign condition allows appropriate reassurance, avoids unnecessary escalation of immunosuppression, and supports shared decision making around therapies that may modestly improve appearance over time.

## Conflicts of interest

None disclosed.

## References

[1] Tolaymat L., Hall M.R. (2026). StatPearls.

[2] Spigariolo C.B., Giacalone S., Nazzaro G. (2021). Pigmented purpuric dermatoses: a complete narrative review. J Clin Med.

[3] Pu Y.J., Jiang H.J., Zhang L. (2023). Purpura annularis telangiectodes of Majocchi: a case report. World J Clin Cases.

[4] Zaldivar Fujigaki J.L., Anjum F. (2026). StatPearls.

[5] Carvajal D., Quiroz C., Morales C., Fernández J. (2019). Granulomatous pigmented purpuric dermatosis: report of a Latin-American case with blaschkoid distribution. An Bras Dermatol.

[6] Alhomieed M.F., Al Hartany L.O., Alghorab M.A. (2025). The effect of pregnancy on dermatological disorders: a systematic review. Clin Pract.

[7] Schober S.M., Peitsch W.K., Bonsmann G. (2014). Early treatment with rutoside and ascorbic acid is highly effective for progressive pigmented purpuric dermatosis. J Dtsch Dermatol Ges.

